# Synthesis, Biological Evaluation, and SAR Studies of 14β-phenylacetyl Substituted 17-cyclopropylmethyl-7, 8-dihydronoroxymorphinones Derivatives: Ligands With Mixed NOP and Opioid Receptor Profile

**DOI:** 10.3389/fpsyt.2018.00430

**Published:** 2018-09-19

**Authors:** Vinod Kumar, Willma E. Polgar, Gerta Cami-Kobeci, Mark P. Thomas, Taline V. Khroyan, Lawrence Toll, Stephen M. Husbands

**Affiliations:** ^1^Department of Pharmaceutical Sciences and Natural Products, Central University of Punjab, Bathinda, India; ^2^SRI International, Menlo Park, CA, United States; ^3^Department of Pharmacy and Pharmacology, University of Bath, Bath, United Kingdom; ^4^Department of Biomedical Sciences, Florida Atlantic University, Boca Raton, FL, United States

**Keywords:** opioid, nociceptin, ORL-1, analgesics, kappa opioid receptor, mu opioid receptors

## Abstract

A series of 14β-acyl substituted 17-cyclopropylmethyl-7,8-dihydronoroxymorphinone compounds has been synthesized and evaluated for affinity and efficacy for mu (MOP), kappa (KOP), and delta (DOP) opioid receptors and nociceptin/orphanin FQ peptide (NOP) receptors. The majority of the new ligands displayed high binding affinities for the three opioid receptors, and moderate affinity for NOP receptors. The affinities for NOP receptors are of particular interest as most classical opioid ligands do not bind to NOP receptors. The predominant activity in the [^35^S]GTPγS assay was partial agonism at each receptor. The results are consistent with our prediction that an appropriate 14β side chain would access a binding site within the NOP receptor and result in substantially higher affinity than displayed by the parent compound naltrexone. Molecular modeling studies, utilizing the recently reported structure of the NOP receptor, are also consistent with this interpretation.

## Introduction

There are three classical opioid receptors mu (MOP), delta (DOP), and kappa (KOP), which play important physiological and pharmacological roles especially in pain regulation. In addition to these, the NOP receptor (earlier ORL1) was identified as a fourth member of the opioid receptor family. This G-protein coupled receptor (1) has significant homology with classical opioid receptors; however none of the endogenous opioid ligands show high affinity to NOP. The endogenous ligand for this receptor, nociceptin/orphanin FQ (N/OFQ) (2, 3) is a 17 amino acid peptide having sequence similarity to the opioid peptides, particularly dynorphin, but it itself does not have high affinity for other opioid receptors. Various early studies indicated that the NOP receptor may play an important role in pain regulation (4), the cardiovascular system (5, 6), opioid tolerance (7), learning and memory (8–10), anorexia (11), anxiety (12), and others (6). However, the development of new therapeutics targeting NOP receptors has not proven easy and it has become clear that the biological actions of NOP receptor ligands vary enormously depending on species, route of administration and dose (13).

For example, the pharmacological action of nociceptin on the perception of pain is not straightforward. Early studies on nociceptin provided mutually contradictory results of either increasing or decreasing perception of pain, depending on dose, site and method of administration (14, 15). Whereas Meunier et al. (3) reported nociceptin induced hyperalgesia in the hot plate test when injected intracerebroventricularly (i.c.v) in mice, Rossi et al. (16) found that i.c.v nociceptin produced a transient hyperalgesia followed by analgesia in the tail flick test in mice. More recent evidence from studies using non-human primates, which may have greater translational validity than studies using rodents, appears to confirm that NOP agonists have analgesic effects comparable to morphine (17–19), though variations in level of response have been reported (20).

Often medicinal chemistry programs aim to develop ligands with ever greater selectivity for a particular target so as to decrease the possibility of side effects. More recently there has been a move to rationally design drugs having a multi receptor affinity profile, recognizing the complexity of many disease states (21, 22). The continued development of Cebranopadol, now in multiple clinical trials is an example of this approach (23, 24). Cebranopadol is a potent, full agonist at both MOP and NOP receptors but is reported to have an improved safety profile over standard MOP receptor agonist analgesics. In a similar vein our groups have been interested in the development of compounds with a mixed affinity profile, including MOP partial agonist/NOP receptor partial agonists and separately MOP partial agonist/KOP partial agonists (25–27). In particular, MOP/NOP partial agonists are expected to be analgesic but with reduced side effect profile, including less respiratory depression, low abuse potential and less tolerance development (27–30).

The orvinol, buprenorphine (**1**) is a partial MOP receptor agonist with modest affinity for the NOP receptor (31). Its efficacy in the treatment of pain may involve a NOP receptor component (32). The close homolog of buprenorphine, BU08028 (**2**) (26, 27), displays significant affinity and partial agonist activity for NOP receptors *in vitro* and SAR from this series of orvinols provides evidence that the region of space occupied by the *t*-butyl group in buprenorphine is key to good NOP receptor activity (26). Subsequently, similar NOP activity was found in the related phenethyl orvinols (**3**) (33) further highlighting the importance of the C20 group in the orvinol series.

The 14β-hydroxymorphinan-6-ones naltrexone (**4**) and naloxone are MOP receptor antagonists used in clinical practice. It is known that substituting the C14-oxygen can have a dramatic effect on the opioid receptor profile of these compounds (34, 35). Thus, while 14-O methyl & ethyl derivatives (36, 37) of naltrexone and naloxone are nonselective opioid receptor antagonists, 14-phenylpropyloxymorphinan-6-ones (38) have shown powerful agonist properties. We have previously reported on cinnamoyl esters of naltrexone as MOP receptor antagonists-partial agonists (39). From molecular modeling studies, it is clear that a suitable substituent attached to the C14-oxygen of naltrexone could access the same region of space as the *t*-butyl group of buprenorphine and it therefore seemed possible that such a series of ligands might display the mixed MOP/NOP receptor partial agonist activity desired.

## Chemistry

**Scheme 1:**
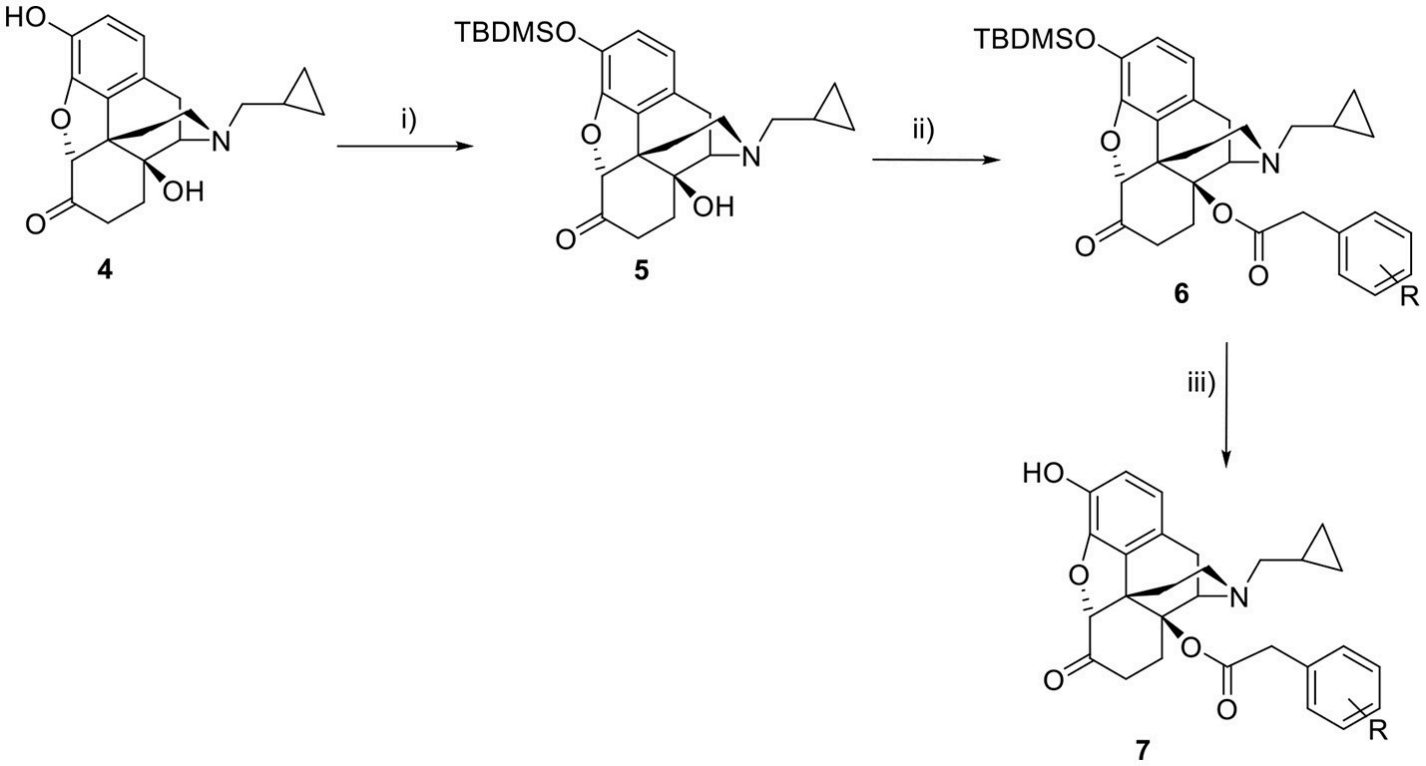
Synthesis of 14-*O*-phenylacetylnaltrexone and analogs. Reagents and conditions: (i) TBDMSiCl, imidazole, DCM, rt; (ii) R-phenylacetic anhydride, toluene, 125°C; (iii) MeOH-HCl (6N) 1:1, reflux.


The 3-hydroxy group of **4** was protected with tert-butyldimethylsilyl chloride in order to carry out selective esterification of the 14-hydroxy group. The tendency of the C6-carbonyl to exist in its enol form meant that clean esterification was not possible with acyl chlorides but could be achieved with the appropriate anhydrides which were synthesized from the corresponding phenylacetic acid and triphosgene. Thereafter the 3-hydroxy group was regenerated using a 1:1 mixture of methanol and HCl (6N) to give the target esters (**7**) (Scheme 1).

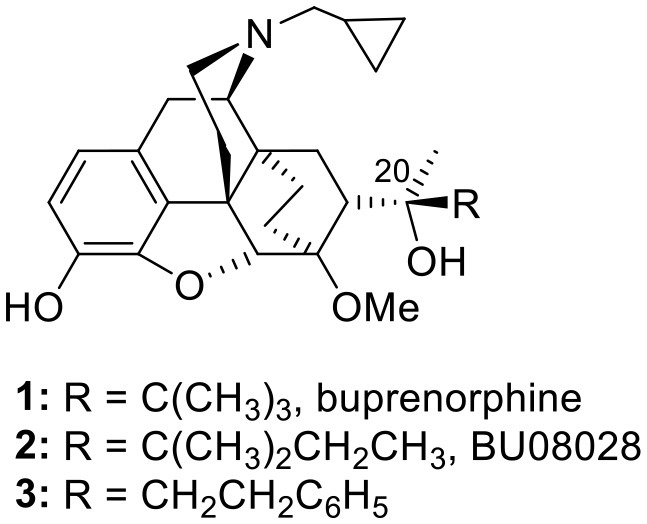


## Results

Affinities for the individual opioid receptors were determined in displacement binding assays in recombinant human opioid receptors transfected into Chinese hamster ovary (CHO) cells as previously described (31). The displaced selective radioligands were [^3^H]N/OFQ (NOP), [^3^H]DAMGO (MOP), [^3^H]Cl-DPDPE (DOP), and [^3^H]U69593 (KOP). All of the ligands displayed high affinity binding in the subnanomolar to nanomolar range toward MOP, KOP, and DOP receptors, with 1–2 orders of magnitude lower affinity at NOP (Table 1). No selectivity in binding between MOP, KOP, and DOP receptors was expected or seen with this series of compounds; similarly there was no substantial effect on the affinities of the ligands at MOP, KOP, and DOP receptors on introduction of a substituent to the aryl ring of the phenylacetyl group. At NOP receptors, it appears that a substituent on the ring may be beneficial to affinity with a two- to four-fold increase in affinity on addition of a single substituent (compare unsubstituted **7a** to substituted analog **7b**-**7k**). When compared with the parent compound **4**, these ligands displayed a substantial increase in binding affinity toward the NOP receptor, a small increase in affinity at DOP (two- to eight-fold) and no change at MOP and KOP. Affinities were almost identical to those of buprenorphine (**1**).

**Table 1:** Binding affinities of new compounds to human opioid receptor transfected into CHO cells^a^.

**Cpd**	**R**	**Ki/nM**			
		**NOP**	**MOP**	**DOP**	**KOP**
**7a**	H	127 ± 17	0.86 ± 0.18	3.10 ± 0.60	1.14 ± 0.46
**7b**	4-CH_3_	36.3 ± 4.2	1.87 ± 0.09	1.70 ± 0.20	1.56 ± 0.28
**7c**	2-CH_3_	44.1 ± 3.1	3.59 ± 0.86	1.97 ± 0.19	1.69 ± 1.2
**7d**	3-CH_3_	49.9 ± 3.5	1.91 ± 0.51	5.58 ± 0.22	2.80 ± 0.91
**7e**	3-OCH_3_	50.1 ± 2.7	1.10 ± 0.08	3.61 ± 0.34	1.44 ± 0.33
**7f**	4-OCH_3_	49.8 ± 7.1	1.08 ± 0.33	2.56 ± 0.48	1.90 ± 0.42
**7g**	3,4-OCH_2_O-	94.3 ± 28	0.99 ± 0.35	1.25 ± 0.20	1.20 ± 0.42
**7h**	2-OCH_3_	62.3 ± 4.9	3.77 ± 0.90	2.12 ± 0.59	3.52 ± 0.90
**7i**	2-F	69.7 ± 2.4	2.59 ± 1.1	4.07 ± 0.91	4.55 ± 0.70
**7j**	4-Cl	51.3 ± 14	1.78 ± 0.03	4.95 ± 1.1	3.95 ± 0.59
**7k**	2-Cl	32.6 ± 2.3	4.66 ± 1.8	3.24 ± 0.15	1.34 ± 0.50
**3**	–	>10K	0.66 ± 0.10	10.7 ± 0.82	1.10 ± 0.22
**1**	–	77.4 ± 16	1.5 ± 0.8	6.1 ± 0.4	2.5 ± 1.2

a *Data are the average ± SD from two experiments, each carried out in triplicate. Tritiated ligands were [^3^H]DAMGO (MOP), [^3^H]N/OFQ (NOP), [^3^H]Cl-DPDPE (DOP), and [^3^H]U69593 (KOP)*.

The *in vitro* assay used to determine opioid receptor functional activity was the [^35^S]GTPγS binding stimulation assay, which, like the binding assays, was performed in human receptor transfected CHO cells as described previously (31). Agonist efficacy at these opioid receptors was determined in comparison to the standard selective agonists N/OFQ (NOP), DAMGO (MOP), DPDPE (DOP), and U69593 (KOP) (Table 2). The ligands were predominantly low efficacy agonists at MOP receptors. **7f**, **7g**, and **7j** were also evaluated as MOP receptor antagonists with **7f** and **7g** proving to be very potent competitive antagonists (pA2 values of 10.58 ± 0.13 and 10.29 ± 0.24, respectively, Table 3), whereas **7j** was non-competitive, as determined by a Schild analysis with a slope different than −1. Similar results were obtained at the other receptors with partial agonism being the standard activity. **7f** had sufficiently low efficacy at KOP, DOP, and NOP receptors to warrant evaluation as an antagonist at each. Whilst competitive at the MOP receptor, inhibition was non-competitive at the other receptors, with IC_50_ values of 12.4 ± 2.25, 12.2 ± 0.11, 48.1± 14.06, and 5,637 ± 2,242 nM at MOP, KOP, DOP, and NOP, respectively.

**Table 2:** Opioid agonist stimulation of [^35^S]GTPγS binding in recombinant human opioid receptor^a^.

	**NOP**		**MOP**		**DOP**		**KOP**	
**Cpd**	**EC_50_/nM**	**% stim**	**EC_50_/nM**	**% stim**	**EC_50_/nM**	**% stim**	**EC_50_/nM**	**% stim**
**7a**	401 ± 161	28.1 ± 4.8	3.8 ± 1.8	31.6 ± 4.2	5.6 ± 0.4	30.5 ± 5.5	1.2 ± 0.6	44.5 ± 13
**7b**	169 ± 3.4	22.3 ± 1.4	1.6 ± 0.9	34.9 ± 2.0	3.8 ± 1.1	27.0 ± 4.8	6.3 ± 2.2	11.5 ± 2.7
**7c**	106 ± 32.2	21.1 ± 1.4	2.2 ± 0.2	41.9 ± 1.4	201 ± 59	12.6 ± 3.1	2.9 ± 1.8	86.3 ± 6.5
**7d**	855 ± 185	59.3 ± 2.4	5.2 ± 2.0	40.1 ± 4.4	21.3 ± 2.2	15.8 ± 0.1	1.0 ± 0.3	46.4 ± 3.6
**7e**	374 ± 81.6	36.1 ± 0.3	13.3 ± 2.2	11.3 ± 1.3	9.9 ± 0.2	35.7 ± 0.7	1.7 ± 0.4	46.3 ± 8.1
**7f**	61.8 ± 20.1	8.9 ± 1.0	^*^	7.8 ± 3.5	59.4 ± 19.6	18.4 ± 1.3	^*^	6.5 ± 3.3
**7g**	562 ± 67.5	43.5 ± 9.4	^*^	12.1 ± 3.7	^*^	----	5.4 ± 0.1	41.1 ± 0.9
**7h**	479 ± 33.3	40.8 ± 5.4	0.5 ± 0.1	37.3 ± 0.7	4.15 ± 1.85	19.2 ± 4.9	1.6 ± 0.3	50.4 ± 6.0
**7i**	94.1 ± 24.7	14.5 ± 2.9	2.0 ± 0.7	27.9 ± 2.8	^*^	----	4.0 ± 2.0	43.8 ± 4.7
**7j**	298 ± 18.6	18.9 ± 2.7	^*^	8.2 ± 7.2	2.91 ± 10.6	37.2 ± 0.2	5.4 ± 1.3	37.1 ± 0.2
**7k**	808 ± 45.8	49.9 ± 0.7	2.4 ± 0.6	39.3 ± 4.7	^*^	----	13.9 ± 6.4	31.3 ± 5.6
**1**	116 ± 88.0	21.0 ± 8.4	10.2 ± 2.2	28.7 ± 1.1	>10,000	----	>10,000	----
DAMGO	----	----	35.3 ± 0.5	100	----	----	----	----
Nociceptin	8.1 ± 1.4	100	----	----		----	----	----
DPDPE	----	----	----	----	6.9 ± 0.4	100	----	----
U69,593	----	----	----	----	----	----	78.5 ± 8.8	100

a *Data are the average ± SD from at least two experiments, each carried out in triplicate*.

* *Too little stimulation (if < 15% an EC_50_ was not always determined)*.

*N/OFQ, Nociceptin/orphinan FQ*.

**Table 3:** Antagonist activity of selected compounds at the MOP receptor in the [^35^S]GTPγS binding assay.

	**MOP**	
**Compound**	**Ke**	**pA2**
**7f**	0.026 ± 0.008	10.58 ± 0.14
**7g**	0.051 ± 0.018	10.29 ± 0.24
**7j**		Non-competitive

*Schild analysis indicated that compound **7j** had a slope significantly different than −1.0. This indicated non-competitive antagonism and a pA2 could not be determined. In an inhibition assay it had an IC_*5*0_ value of 5.9 ± 1.84 nM when inhibiting DAMGO stimulation of [^*3*5^S]GTPγS binding. Results represent cumulative data from at least three separate experiments*.

**Figure 1:**
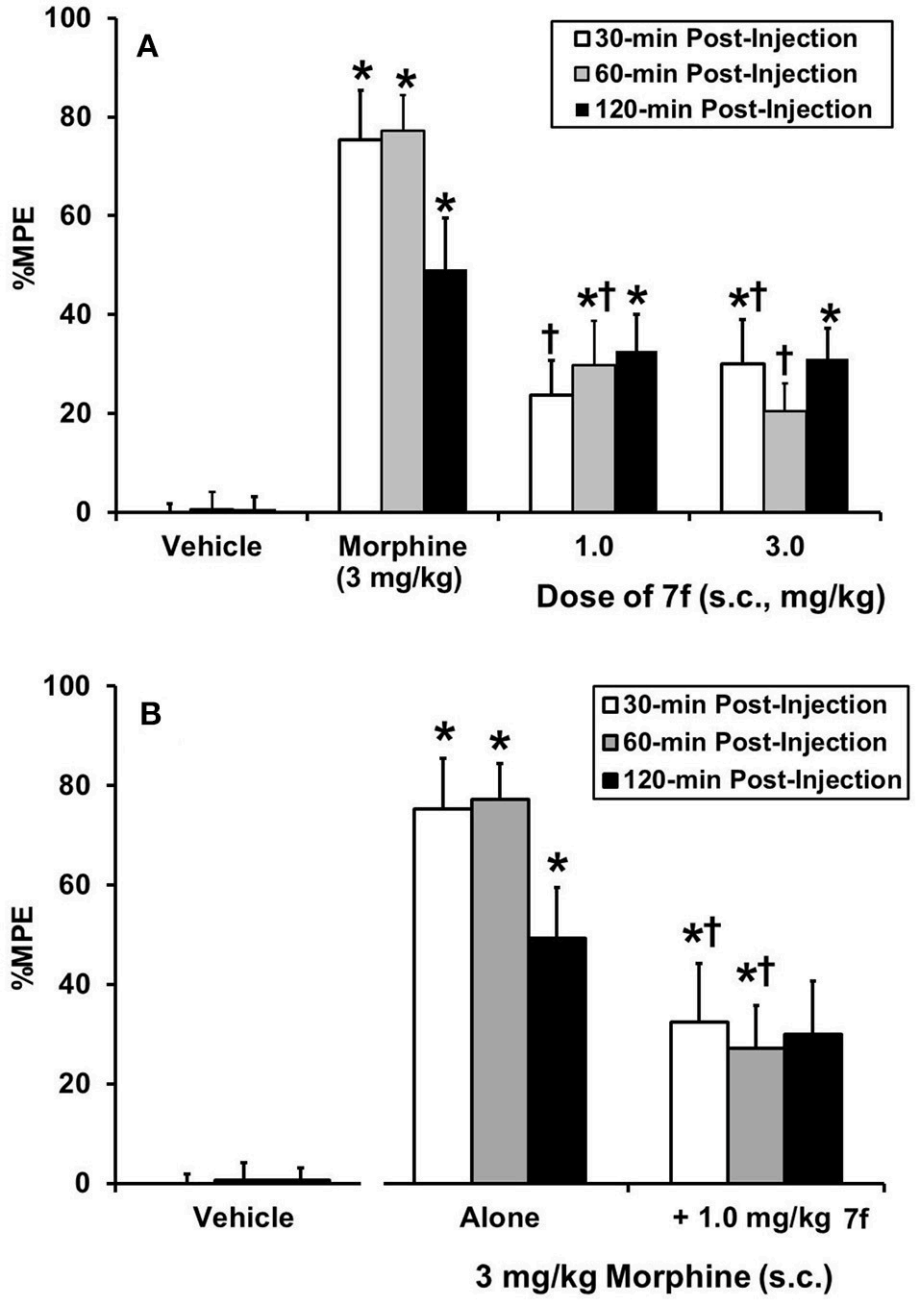
Acute thermal antinociceptive effect of **7f** alone **(A)** or as a pretreatment to morphine **(B)**, using the mouse tail flick assay (*N* = 8/group). Data are mean %MPE (± SEM). ^*^Significant difference from vehicle control; †Significant difference from morphine alone (*P* < 0.05). Behavioral results were analyzed by use of repeated measures ANOVAs with drug treatment (**7f**, morphine) as between group variables and post-injection time (30, 60, and 120 min) as the repeated measure followed by Tukey/Kramer *post-hoc* tests where appropriate.

**7f** was evaluated in CD1 mice using the tail flick assay with an analgesia instrument (Stoetling) that uses radiant heat. Methods were as reported previously (27, 31). The overall ANOVA indicated that there was a significant interaction effect [*F*_(6, 56)_ = 3.96, *P* < 0.05]. The positive control morphine (3 mg/kg) produced the anticipated increase in %MPE at all time points. At the doses tested (1 and 3 mg/kg) **7f** produced low levels of antinociception, consistent with partial agonist activity demonstrated in the [^35^S]GTPγS binding assay. The 1 mg/kg dose of **7f** produced a significant increase in tail flick latency compared to vehicle controls at the 60- and 120-min time points, whereas the 3.0 mg/kg dose produced significant antinociception at the 30- and 120-min time points (Figure 1A). Given that both doses of **7f** produced similar levels of antinociception, we examined whether the lower dose would alter morphine-induced analgesia. As evident in Figure 1B, when **7f** was given as a pretreatment to morphine, morphine-induced antinociception was attenuated at the 30- and 60-min time points (*P* < 0.05).

## Discussion

Substitution at 14-*O* position of naltrexone (**4**) has a significant impact on the pharmacological profile. Lia et al. (40) reported on a series of 14-*O* heterocyclic esters of **4** as selective MOP receptor antagonists with subnanomolar to nanomolar binding affinities. Similarly we have reported (39) that the predominant activity of 14-*O* cinnamoyl esters of **4** was MOP partial agonism/antagonism both *in vitro* and *in vivo*. In contrast, the equivalent phenylpropyl ether was a potent agonist in a battery of thermal nociceptive assays (38); thus substitution at the 14-*O* position of **4** plays a critical role in modulating activity, and predominantly efficacy, of the ligands at the traditional opioid receptors MOP, DOP, and KOP. In the current study, this SAR is further explored and extended to include activity at the NOP receptor. The new ligands, substituted 14-*O*-phenylacetyl esters of **4**, were evaluated for binding affinities and efficacies at MOP, DOP, and KOP, and NOP receptors. Phenylacetyl substitution at the 14-oxygen had little effect on affinity at MOP, DOP, and KOP receptors, but did substantially increase the binding affinity at NOP receptors. Addition of a substituent to the aryl ring of the phenylacetyl group further increased affinity for NOP receptors leading to a series of compounds with binding profiles directly comparable to buprenorphine (**1**). This provides support for our hypothesis that the group, in this case phenylacetyl, attached to the 14-*O* of **4** can access the same space as the *t*-butyl group in **1**, leading to moderate affinity at NOP receptors. The non-competitive binding seen with **7f** and **7j** may relate to the increased lipophilicity of these esters relative to **4**. The calculated logPs of **7j** (logP 4.41 ± 0.57) and **7f** (3.73 ± 0.57) (calculated using ACD/I-lab 2.0) are similar to those found with the orvinols—a series for which there is evidence for pseudo-irreversible binding in *in vitro* bioassays (25, 41).

**Figure 2:**
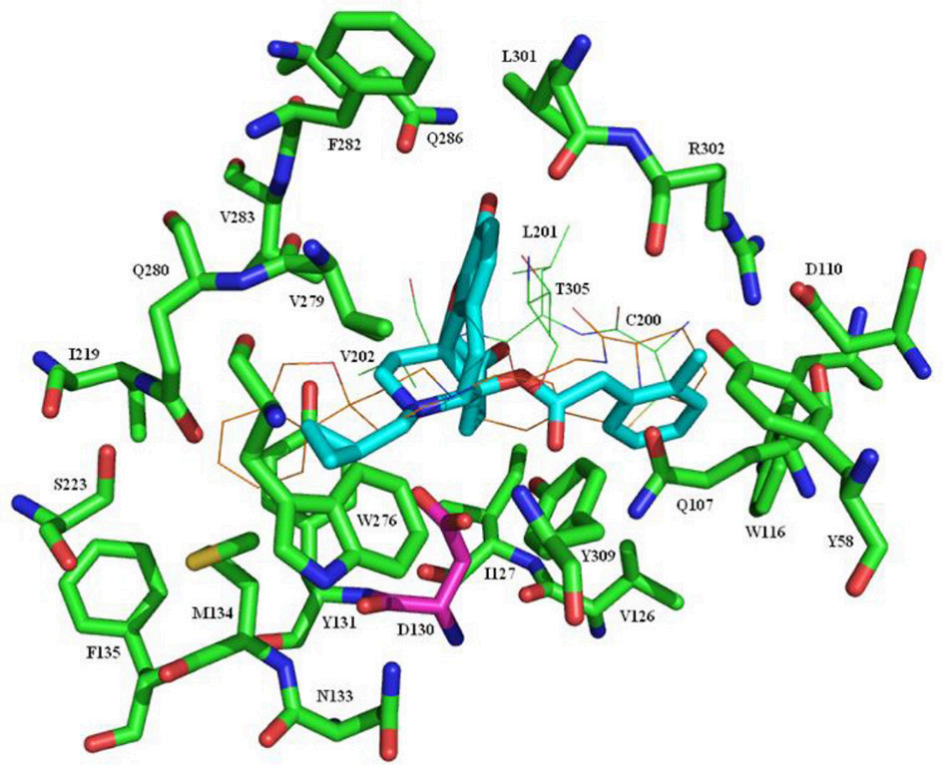
Docking of **7c** in the binding site of the NOP receptor. **7c** is shown with cyan carbons. The protein is shown with green carbons apart from D130 which has purple carbons. For clarity, residues C200, L201, V202, and T305 are shown as lines rather than sticks. The crystal structure ligand, C-24, is shown as lines with brown carbons.

**Figure 3:**
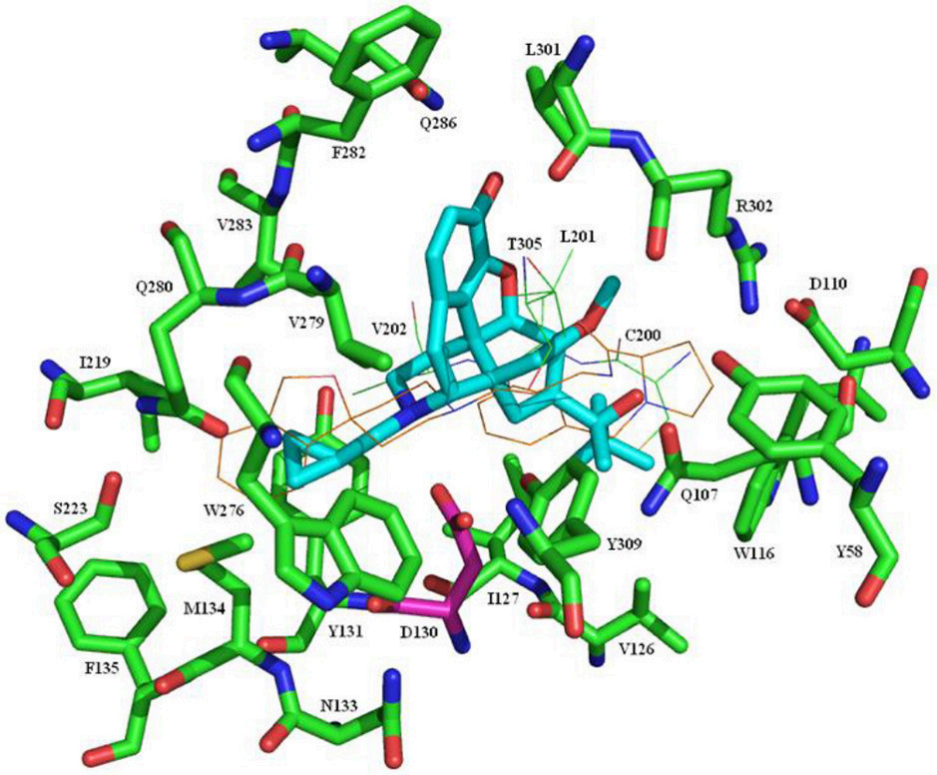
Docking of Buprenorphine (**1**) in the binding site of the NOP receptor. **1** is shown with cyan carbons. The protein is shown with green carbons apart from D130 which has purple carbons. For clarity, residues C200, L201, V202, and T305 are shown as lines rather than sticks. The crystal structure ligand, C-24, is shown as lines with brown carbons.

Recently the structure of the NOP receptor in complex with the peptide mimetic C-24 has been determined (42). As part of the current study, **7c** was docked to the binding site of the crystal structure using GOLD. The docked pose of **7c** that best fit with the known interactions of C-24 with the protein is illustrated in Figure 2. Key interactions are between the basic nitrogen and Asp130, while the cyclopropylmethyl group occupies, but not fully, a lipophilic site accessed by the dihydroisobenzofuran head group of C-24. Most interestingly, the phenylacetyl type side chain of **7c** extends into the same region occupied by the pyrolidine ring of C-24 (including the amino acid residues Gln107, Asp110, Trp116, and Val126) and perhaps explains the substantial increase in affinity for these new ligands relative to the parent compound **3**, which cannot access this region. The Schrödinger software was then used to superimpose buprenorphine on the minimized structure of **7c** in the protein-ligand complex resulting in the same interactions between the basic nitrogen and the cyclopropylmethyl group with the protein and now with the bulky *t*-butyl group accessing the same region as the phenylacetyl group of **7c** (Figure 3). We have shown previously that minor changes to the *t*-butyl group of **1** can have a significant impact on binding affinity and efficacy at the NOP receptor (26, 27) and again, the interaction of this group with the site defined by, amongst other residues, Gln107, Asp110, Trp116, and Val126 could explain this finding. This docking pose would also help explain the lack of effect on NOP affinity on substituting the aromatic A-ring of **1** with halogens (26) as the A-ring extends into a very large, open region of the binding pocket, making no close interactions with receptor residues.

The predominant activity in the [^35^S]GTPγS assay was of partial agonism at each of the receptors under study. Thus, when compared to the parent compound **4**, an antagonist, introduction of the phenylacetyl side chain has increased efficacy at each receptor. The effect was most pronounced at the KOP where one compound, **7c**, had high efficacy (86% of the standard) and a number of others fell in the 40–50% range. In this assay, efficacies were somewhat lower at MOP and lower still at DOP receptors. Some consistent SAR does emerge, with *ortho* substitution tending to give the highest efficacy, followed by *meta* and then *para* at both MOP and KOP receptors. A similar trend is observed at NOP receptors, where the *ortho* and *meta*-substituted ligands were typically higher efficacy than their *para* substituted equivalents. At MOP and NOP receptors a number of the new ligands had profiles somewhat similar to **1**, though typically with more selectivity for MOP. The most substantial difference to **1** was at the KOP receptor where the potent partial agonism of many of the current series contrasts with the potent antagonism characteristic of **1**. Compared to the closely related cinnamoyl esters reported previously (39), these phenylacetyl esters have similar affinities, but higher efficacies at MOP, DOP, and KOP receptors (NOP receptor activity was not measured for the cinnamoyl esters).

The 4-methoxy substituted analog **7f** was of interest due to its good affinity and very low level stimulation of all the receptors in the [^35^S]GTPγS assay. Agonists for KOP and DOP receptors have been, and continue to be, evaluated as potential analgesics either as selective ligands (43) or dual-acting (44). In an extension of the argument made earlier for the development of mixed MOP/NOP agonists, it could be envisaged that ligands displaying low efficacy at each of the receptors might provide analgesia with very little in the way of side-effect profile. The low level of antinociception observed in the mouse tail flick assay and the ability to act as a morphine antagonist is consistent with this hypothesis and **7f** may provide a useful lead in the development of new, safer analgesics.

## Conclusion

The hypothesis that introduction of a lipophilic group to the 14-oxygen of **4** would introduce NOP receptor affinity has been validated by the present study. Moderate affinity, equivalent to that of the orvinol buprenorphine (**1**), was seen alongside low efficacy partial agonism, supporting our belief that the *t*-butyl group of **1** and the phenylacetyl group of the current series might access the same region of the NOP receptor. This is reinforced by docking studies, to the recently solved crystal structure of the NOP receptor that provide a rationale for the moderate affinity shown by the ligands reported here and also, help explain the SAR of close analogs of **1** (26). As expected, introduction of the 14-*O* side chain also raised efficacy relative to **4** at the standard opioid receptors.

## Methods

Reagents and solvents were purchased from Sigma-Aldrich or Alfa Aesar and used as received. ^1^H and ^13^C NMR spectra were obtained with a Brucker-400-MHz instrument (^1^H at 400 MHz, ^13^C at 100 MHz); δ in ppm, *J* in Hz with TMS as an internal standard. ESIMS: microTOF (BRUKER). Microanalysis: Perkin-Elmer 240C analyzer. Column Chromatography was performed using pre-packed column in combi flash instrument. Ligands were tested as their hydrochloride salts, prepared by adding 5 equivalent of HCl (1 N solution in diethyl ether) in a solution of compound in anhydrous methanol. All reactions were carried out under an inert atmosphere of nitrogen unless otherwise indicated. All compounds were >95% pure.

### General procedure. 14-*O*-esterification

To a solution of TBDMS-protected naltrexone (**5**) (0.4 mmol) in anhydrous toluene, optionally substituted phenylacetic anhydride (0.8 mmol), and DMAP (0.04 mmol) were added and the reaction mixture refluxed for 16 h. After completion saturated sodium bicarbonate (15 mL) was added and the aqueous layer extracted with EtOAc (3 × 10 mL). Organic layer was washed with water (2 × 20 mL), brine (10 mL) and dried over magnesium sulfate and evaporated *in vacuo* to obtain crude product which was purified by flash chromatography using methanol:dichloromethane (0.5:99.5).

### General procedure. TBDMS deprotection

The substrate (0.3 g) was dissolved in 6 mL (1:1) solution of methanol:hydrochloric acid (6N) and refluxed for 5 h. The reaction mixture was cooled to 0°C and neutralized with saturated aq. sodium bicarbonate. The organic layer was extracted with ethyl acetate (3 × 20 ml), washed with water (2 × 25 mL), brine (25 mL), dried over magnesium sulfate and evaporated to obtained crude product which was purified by flash chromatography using methanol:dichloromethane:ammonium hydroxide (2:97.5:0.5).

### 14β-phenylacetyl-17-cyclopropylmethyl-7,8-dihydronoroxymorphinone (7a)

White Solid; ^1^H NMR (CDCl_3_) δ 0.09–0.12 (2H, m), 0.51–0.54 (2H, m), 0.78–0.83 (1H, m), 1.41–1.45 (1H, m), 1.57 (1H, dt, *J* = *3.72* and *14.44Hz*), 2.09–2.21 (2H, m), 2.23–2.52 (4H, m), 2.58–2.67 (2H, m), 2.79–2.82 (1H, m), 3.07 (1H, d, *J* = *18.2 Hz*), 3.77 (2H, m), 4.44 (1H, d, *J* = *5.52 Hz*), 4.51 (1H, s), 6.59 (1H, d, *J* = *8.0 Hz*), 6.72 (1H, d, *J* = *8.0 Hz*), 7.24–7.31 (2H, m), 7.36–7.41 (3H, m); HRMS, m/z for (C_28_H_30_NO_5_) [MH]^+^, calcd- 460.2124, found- 460.2103.

### 14β-(4′-methylphenylacetyl)-17-cyclopropylmethyl-7,8-dihydronoroxymorphinone (7b)

White Solid; ^1^H NMR (CDCl_3_) δ 0.09–0.12 (2H, m), 0.50–0.54 (2H, m), 0.74–0.80 (1H, m), 1.44–1.48 (1H, m), 1.61 (1H, dt, *J* = *3.72* and *14.44Hz*), 2.14–2.22 (2H, m), 2.27–2.51 (8H, m), 2.57–2.61 (1H, m), 2.78–2.82 (1H, m), 3.07 (1H, d, *J* = *18.2 Hz*), 3.74–3.76 (2H, m), 4.48 (1H, d, *J* = *5.52 Hz*), 4.53 (1H, s), 6.60 (1H, d, *J* = *8.0 Hz*), 6.72 (1H, d, *J* = *8.0 Hz*), 7.16 (2H, d, *J* = *8.0 Hz*), 7.27 (2H, d, *J* = *8.0 Hz*); HRMS, m/z for (C_29_H_32_NO_5_) [MH]^+^, calcd-474.2280, found-474.2329.

### 14β-(2′-methylphenylacetyl)-17-cyclopropylmethyl-7,8-dihydronoroxymorphinone (7c)

White Solid; ^1^H NMR (CDCl_3_) δ 0.09–0.14 (2H, m), 0.51–0.55 (2H, m), 0.78–0.84 (1H, m), 1.32–1.35 (1H, m), 1.51 (1H, dt, *J* = *3.72* and *14.44Hz*), 2.10–2.50 (10H, m), 2.62–2.68 (1H, m), 2.74–2.81 (1H, m), 3.03 (1H, d, *J* = *18.2 Hz*), 3.76–3.88 (2H, m), 4.42 (1H, s), 4.51 (1H, d, *J* = *5.52 Hz*), 5.90 (1H, bd), 6.59 (1H, d, *J* = *8.0 Hz*), 6.72 (1H, d, *J* = *8.0 Hz*), 7.20–7.23 (3H, m), 7.29–7.33 (1H, m); HRMS, m/z for (C_29_H_32_NO_5_) [MH]^+^, calcd- 474.2280, found- 474.2258.

### 14β-(3′-methylphenylacetyl)-17-cyclopropylmethyl-7,8-dihydronoroxymorphinone (7d)

White Solid; ^1^H NMR (CDCl_3_) δ 0.09–0.12 (2H, m), 0.50–0.0.54 (2H, m), 0.70–0.75 (1H, m), 1.45–1.49 (1H, dd, *J* = *4.00* and *12.1 Hz*), 1.59–1.66 (1H, dt, *J* = *3.72* and *14.44Hz*), 2.14–2.31 (2H, m), 2.32–2.40 (8H, m), 2.67 (1H, dd, *J* = *4.0* and *12.1 Hz*), 2.76–2.81 (1H, m), 3.07 (1H, d, *J* = *18.4 Hz*), 3.69 (2H, dd, *J* = *8.0* and *18.4 Hz*), 4.49 (1H, d, *J* = *4.0 Hz*), 4.53 (1H, s), 5.75 (1H, bd), 6.60 (1H, d, *J* = *8.0 Hz*), 6.73 (1H, d, *J* = *8.0 Hz*), 7.11 (1H, d, *J* = *6.1 Hz*), 7.17 (3H, d, *J* = *8.1 Hz*); HRMS, m/z for (C_29_H_32_NO_5_) [MH]^+^, calcd- 474.2280, found- 474.2288.

### 14β-(3′-methoxyphenylacetyl)-17-cyclopropylmethyl-7,8-dihydronoroxymorphinone (7e)

White Solid; ^1^H NMR (CDCl_3_) δ 0.09–0.14 (2H, m), 0.50–0.0.54 (2H, m), 0.69–0.74 (1H, m), 1.45–1.49 (1H, dd, *J* = *4.00* and *12.1 Hz*), 1.59–1.66 (1H, dt, *J* = *3.72* and *14.44Hz*), 2.14–2.31 (2H, m), 2.31–2.41 (5H, m), 2.67 (1H, dd, *J* = *4.0* and 12.1 *Hz*), 2.76–2.81 (1H, m), 3.07 (1H, d, *J* = *18.4 Hz*), 3.71 (2H, dd, *J* = *8.0* and *18.4 Hz*), 3.83 (3H, s), 4.49 (1H, d, *J* = *4.0 Hz*), 4.54 (1H, s), 5.79 (1H, bd), 6.60 (1H, d, *J* = *8.0 Hz*), 6.73 (1H, d, *J* = *8.0 Hz*), 6.83 (1H, dd, *J* = *4.0* and 8.1 *Hz*), 6.95–6.99 (2H, m), 7.28–7.30 (1H, m); HRMS, m/z for (C_29_H_32_NO_6_) [MH]^+^, calcd- 490.2230, found- 490.2278.

### 14β-(4′-methoxyphenylacetyl)-17-cyclopropylmethyl-7,8-dihydronoroxymorphinone (7f)

White Solid; ^1^H NMR (CDCl_3_) δ 0.05–0.08 (2H, m), 0.46–0.50 (2H, m), 0.70–0.73 (1H, m), 1.42–1.45 (1H, m), 1.56 (1H, dt, *J* = *3.76* and *14.44Hz*), 2.11–2.16 (2H, m), 2.18–2.47 (5H, m), 2.61–2.67 (1H, m), 2.74–2.79 (1H, m), 3.03 (1H, d, *J* = *18.2 Hz*), 3.64–3.73 (2H, m), 3.79 (3H, s), 4.44 (1H, d, *J* = *5.52 Hz*), 4.52 (1H, s), 6.56 (1H, d, *J* = *8.0 Hz*), 6.69 (1H, d, *J* = *8.0 Hz*), 6.85 (2H, d, *J* = *8.0 Hz*), 7.25 (2H, d, *J* = *8.0 Hz*); HRMS, m/z for (C_29_H_32_NO_6_) [MH]^+^, calcd- 490.2230, found- 490.2200.

### 14β-(3′,4′-dioxymethylenephenylacetyl)-17-cyclopropylmethyl-7,8-dihydronoroxymorphinone (7g)

White Solid; ^1^H NMR (CDCl_3_) δ 0.09–0.14 (2H, m), 0.51–0.54 (2H, m), 0.89–0.95 (1H, m), 1.46–1.51 (1H, m), 1.57 (1H, dt, *J* = *3.76* and *14.44Hz*), 2.11–2.31 (3H, m), 2.36–2.46 (4H, m), 2.65–2.72 (1H, m), 2.76–2.82 (1H, m), 3.08 (1H, d, *J* = *18.2 Hz*), 3.68–3.72 (2H, m), 4.49 (1H, d, *J* = *5.52 Hz*), 4.58 (1H, s), 5.50 (1H, bd), 5.97 (2H, s), 6.61 (1H, d, *J* = *8.0 Hz*), 6.73 (1H, d, *J* = *8.0 Hz*), 6.79–6.82 (2H, m), 6.92 (1H, s); HRMS, m/z for (C_29_H_30_NO_7_) [MH]^+^, calcd- 504.2022, found- 504.2069.

### 14β-(2′-methoxyphenylacetyl)-17-cyclopropylmethyl-7,8-dihydronoroxymorphinone (7h)

White Solid; ^1^H NMR (CDCl_3_) δ 0.08–0.11 (2H, m), 0.49–0.52 (2H, m), 0.78–0.84 (1H, m), 1.32–1.35 (1H, m), 1.51 (1H, dt, *J* = *3.72* and *14.44Hz*), 2.10–2.45 (6H, m), 2.55–2.65 (2H, m), 2.74–2.81 (1H, m), 3.03 (1H, d, *J* = *18.2 Hz*), 3.76 (2H, m), 3.81 (3H, s), 4.34 (1H, s), 4.43 (1H, d, *J* = *5.52 Hz*), 5.61 (1H, bd), 6.55 (1H, d, *J* = *8.0 Hz*), 6.68 (1H, d, *J* = *8.0 Hz*), 6.89 (1H, d, *J* = *8.0 Hz*), 6.93 (1H, m), 7.24–7.28 (2H, m); HRMS, m/z for (C_29_H_32_NO_6_) [MH]^+^, calcd- 490.2230, found- 490.2228.

### 14β-(2′-fluorophenylacetyl)-17-cyclopropylmethyl-7,8-dihydronoroxymorphinone (7i)

White Solid; ^1^H NMR (CDCl_3_) δ 0.06–0.09 (2H, m), 0.48–0.0.51 (2H, m), 0.71–0.75 (1H, m), 1.33–1.36 (1H, m), 1.56–1.64 (1H, dt, *J* = *3.72* and *14.44Hz*), 2.04–2.42 (6H, m), 2.46–2.62 (2H, m), 2.76–2.80 (1H, m), 3.07 (1H, d, *J* = *18.4 Hz*), 3.72 (2H, m), 4.42 (1H, d, *J* = *4.0 Hz*), 4.44 (1H, s), 5.65 (1H, bd), 6.56 (1H, d, *J* = *8.0 Hz*), 6.68 (1H, d, *J* = *8.0 Hz*), 7.08–7.14 (2H, m), 7.25–7.29 (1H, m) 7.35–7.37 (1H, m); HRMS, m/z for (C_28_H_29_FNO_5_) [MH]^+^, calcd- 478.2030, found- 478.2073.

### 14β-(4′-chlorophenylacetyl)-17-cyclopropylmethyl-7,8-dihydronoroxymorphinone (7j)

White Solid; ^1^H NMR (CDCl_3_) δ 0.04–0.10 (2H, m), 0.47–0.50 (2H, m), 0.65–0.69 (1H, m), 1.43–1.46 (1H, m), 1.59 (1H, dt, *J* = *3.72* and *14.44Hz*), 2.08–2.16 (1H, m), 2.21–2.34 (4H, m), 2.39–2.48 (2H, m), 2.62–2.65 (1H, m), 2.74–2.81 (1H, m), 3.04 (1H, d, *J* = *18.2 Hz*), 3.72–3.74 (2H, m), 4.43 (1H, d, *J* = *5.52 Hz*), 4.53 (1H, s), 6.56 (1H, d, *J* = *8.0 Hz*), 6.70 (1H, d, *J* = *8.0 Hz*), 7.29–7.31 (4H, m); HRMS, m/z for (C_28_H_29_ClNO_5_) [MH]^+^, calcd- 494.1734, found- 494.1734.

### 14β-(2′-chlorophenylacetyl)-17-cyclopropylmethyl-7,8-dihydronoroxymorphinone (7k)

White Solid; ^1^H NMR (CDCl_3_) δ 0.08–0.10 (2H, m), 0.49–0.53 (2H, m), 0.76–0.81 (1H, m), 1.30–1.33 (1H, m), 1.56 (1H, dt, *J* = *3.72* and *14.44Hz*), 2.04–2.09 (2H, m), 2.25–2.32 (3H, m), 2.41–2.46 (1H, m), 2.53–2.59 (2H, m), 2.76–2.81 (1H, m), 3.03 (1H, d, *J* = *18.2 Hz*), 3.90 (2H, m), 4.41 (1H, d, *J* = *4.0 Hz*), 4.43 (1H, s), 5.48 (1H, bd), 6.55 (1H, d, *J* = *8.0 Hz*), 6.68 (1H, d, *J* = *8.0 Hz*), 7.24–7.26 (2H, m), 7.40–7.43 (2H, m); HRMS, m/z for (C_28_H_29_ClNO_5_) [MH]^+^, calcd- 494.1734, found- 494.1729.

#### Molecular modeling methods

The 4EA3 crystal structure (42) of NOP was the starting point. The structure was run through the Protein Preparation Wizard in the Schrödinger software suite running under Maestro version 9.3.023. Buprenorphine (**1**) and **7c** were built and minimized using the same software. Both ligands were docked into the binding site using GOLD. The docked pose of **7c** that seemed to best fit with the known interactions of the ligand with the protein was subjected to 1,000 rounds of minimization using the Schrödinger MacroModel software with the constraint that the ligand nitrogen be 2.8 Å from the nearest acidic oxygen of D130. GOLD failed to bind **1** with a sensible pose so the Schrödinger software was used to superimpose **1** on the minimized structure of **7c** in the protein-ligand complex. The protein-**1** complex was then subjected to 1,000 rounds of minimisation. Figures were prepared using PyMOL.

#### *In vitro* characterization

##### Cell culture

All receptors were individually expressed in CHO cells stably transfected with human receptor cDNA, The cells were grown in Dulbecco's Modified Eagle Medium (DMEM) with 10% fetal bovine serum, in the presence of 0.4 mg/ml G418 and 0.1% penicillin/streptomycin, in 100-mm polystyrene culture dishes. For binding assays, the cells were scraped off the plate at confluence. Receptor expression levels were 1.2, 1.6, 1.8, and 3.7 pmol per mg protein for the NOP, MOP, KOP, and DOP receptors, respectively.

##### Receptor binding

Binding to cell membranes was conducted in a 96-well format, as described previously (45, 46). Briefly, cells were removed from the plates, homogenized in 50 mM Tris pH 7.5, using a Polytron homogenizer, then centrifuged once and washed by an additional centrifugation at 27,000 × g for 15 min. The final pellet was re suspended in Tris, and the suspension incubated with [^3^H]DAMGO (51 Ci/mmol, 1.6 nM), [^3^H]Cl-DPDPE (42 Ci/mmol, 1.4 nM), [^3^H]U69593 (41.7 Ci/mmol, 1.9 nM), or [^3^H]N/OFQ (120 Ci/mmol, 0.2 nM) for binding to, MOP, DOP, KOP, and NOP receptors, respectively. Non-specific binding was determined with 1 μM of unlabeled DAMGO ([D-Ala2, N-MePhe4, Gly-ol]-enkephalin), DPDPE ([D-Pen2,D-Pen5]Enkephalin), ethylketocyclazocine, and N/OFQ, respectively. Samples were incubated for 60 min at 25°C in a total volume of 1.0 ml, with 15 μg protein per well. The reaction was terminated by filtration using a Tomtec 96 harvester (Orange, CT) through glass fiber filters and radioactivity was counted on a Pharmacia Biotech beta-plate liquid scintillation counter (Piscataway, NJ). IC_50_ values were calculated using Graphpad/Prism (ISI, San Diego, CA) and Ki values were determined by the method of Cheng and Prusoff (47).

##### [^*3*5^S]GTPγS binding

[^35^S]GTPγS binding was conducted basically as described by Traynor and Nahorski (48). Cells were scraped from tissue culture dishes into 20 mM Hepes, 1 mM EDTA, then centrifuged at 500x *g* for 10 min. Cells were resuspended in this buffer and homogenized using a Polytron Homogenizer. The homogenate was centrifuged at 27,000 × g for 15 min, and the pellet re suspended in Buffer A, containing: 20 mM Hepes, 10 mM MgCl_2_, 100 mM NaCl, pH 7.4. The suspension was re centrifuged at 27,000 × g and suspended once more in Buffer A. For the binding assay, membranes (8–15 μg protein) were incubated with [^35^S]GTPγS (50 pM), GDP (10 μM), and the appropriate compound, in a total volume of 1.0 ml, for 60 min at 25°C. Samples were filtered over glass fiber filters and counted as described for the binding assays. Statistical analysis was conducted using the program Prism. For the antagonist assay, various concentrations of 7f, 7g, and 7j were incubated in the presence of 100 nM N/OFQ, or 1 μM DAMGO, DPDPE or U69593 to determine antagonist potency at NOP, MOP, DOP, and KOP receptors, respectively. Schild analysis was also conducted at MOP receptors using various concentrations of the inhibitor in the present of a full DAMGO dose response curve.

#### *In vivo* testing

##### Animals

Male ICR mice weighing 25–30 g at the start of the experiment were used. Animals were group-housed (*N* = 10/cage) under standard laboratory conditions using nestlets as environmental enrichment in their cages and were kept on a 12:12-hr day/night cycle (lights on at 7:00 a.m.). Testing was conducted during the animals' light cycle between 9 a.m. and 2 p.m. Animals were handled for 3–4 days before the experiments were conducted. On behavioral test days, animals were transported to the testing room and acclimated to the environment for 1 h. Mice were maintained in accordance with the guidelines of SRI International and of the Guidelines for the Care and Use of Mammals in Neuroscience and Behavioral Research (49). Prior to any *in vivo* testing, approval for the behavioral protocols was obtained from the institutional ACUC of SRI International.

##### Drugs

7f was dissolved in 1–2% Dulbecco's modified Eagle's medium and 0.5% aqueous hydroxypropyl cellulose. Morphine hydrochloride (Eli Lilly & Co., Indianapolis, IN) was dissolved in water. Drugs were injected subcutaneously (s.c.) in a volume of 0.1 ml/30 g. Controls received 0.1 ml/30 g of the appropriate vehicle.

##### Assessment of acute thermal nociception

*Tail-flick assay*. Acute nociception was assessed using the tail flick assay with an analgesia instrument (Stoelting) that uses radiant heat. This instrument is equipped with an automatic quantification of tail flick latency, and a 15 s cutoff to prevent damage to the animal's tail. During testing, the focused beam of light was applied to the lower half of the animal's tail, and tail flick latency was recorded.

Baseline values for tail flick latency were determined before drug administration in each animal. The mean basal tail flick latency was 5.39 ± 0.09 SEM. After baseline measures, animals received a subcutaneous injection of their assigned dose of drug(s) and were tested for tail-flick latencies at 30, 60, and 120 min following the last drug injection. Controls received vehicle prior to testing.

*Drug regimen*. In the first series of experiments, animals (*N* = 8/group) received injections of **7f** (1 and 3 mg/kg s.c.) or morphine (3 mg/kg). Given that both 1 and 3 mg/kg **7f** produced similar levels of antinociception, we chose to examine the effects of 1 mg/kg **7f** given as a pretreatment to morphine. In these experiments, animals received 1 mg/kg **7f** or vehicle and 10 min later received an injection of morphine. A group of animals served as vehicle controls. Testing was conducted as described above.

*Statistical analyses*. Antinociception (% maximum potential effect; % MPE) was quantified by the following formula: % MPE = 100[(test latency–baseline latency)/(15–baseline latency)]. If the animal did not respond before the 15-s cutoff, the animal was assigned a score of 100%. Behavioral results were analyzed by use of repeated measures ANOVAs with drug treatment **(7f**, morphine) as between group variables and post-injection time (30, 60, and 120 min) as the repeated measure followed by Tukey/Kramer *post-hoc* tests where appropriate. The level of significance was set at *P* = 0.05.

## Author contributions

SH, LT, and TK participated in the research design. TK, VK, GC-K, MT, and WP conducted the experimental work. SH wrote the manuscript with input from all co-authors.

### Conflict of interest statement

SH and LT are joint inventors on a patent application containing these compounds. The remaining authors declare that the research was conducted in the absence of any commercial or financial relationships that could be construed as a potential conflict of interest.

## Abbreviations

MOP receptor: mu opioid receptor
NOP receptor: nociceptin/orphanin FQ receptor
DOP receptor: delta opioid receptor
KOP receptor: kappa opioid receptor
MPE: maximum percent effect
ANOVA: analysis of variance
DAMGO: [D-Ala2, N-MePhe4, Gly-ol]-enkephalin
DPDPE: [D-Pen2,D-Pen5]enkephalin.
